# Effectiveness and economic evaluation of rhTPO and rhIL-11 in the treatment of cancer therapy induced thrombocytopenia based on real-world research

**DOI:** 10.3389/fphar.2024.1288964

**Published:** 2024-01-23

**Authors:** Fa-Min Gong, Fu-Yue Liu, Xue Ma, Song-Tao Ma, Hong-Tao Xiao, Gang Jiang, Ting-Ting Qi

**Affiliations:** ^1^ Department of Pharmacy, Cancer Hospital Affiliated to University of Electronic Science and Technology, Sichuan Cancer Center, Sichuan Cancer Hospital and Institute, Chengdu, Sichuan, China; ^2^ State Key Laboratory of Biotherapy and Cancer Center, West China Hospital, Sichuan University, Collaborative Innovation Center, Chengdu, Sichuan, China; ^3^ Department of Pharmacy, Chongqing General Hospital, Chongqing, China; ^4^ Department of Pharmacy, Renshou County People’s Hospital, Meishan, Sichuan, China; ^5^ School of Pharmacy, Chengdu Medical College, Chengdu, Sichuan, China

**Keywords:** cancer therapy induced thrombocytopenia (CTIT), recombinant human thrombopoietin (rhTPO), recombinant human interleukin-11 (rhIL-11), effectiveness, economic evaluation

## Abstract

**Objective:** Based on real-world research, we aimed to evaluate the effectiveness and economy of recombinant human thrombopoietin (rhTPO) and recombinant human interleukin 11 (rhIL-11) in the treatment of cancer therapy induced thrombocytopenia (CTIT).

**Methods:** We retrospectively collected clinical data of patients with CTIT who were treated with rhTPO or rhIL-11 in a single cancer hospital from January 2020 to December 2021. Propensity score matching (PSM) was applied to eliminate confounding factors. The measurements of effectiveness analysis were the platelet compliance rate, days of medication, days of compliance, highest platelet count after medication, platelet count elevation before and after medication, and the lowest platelet count after next-cycle cancer therapy. The economic evaluation was performed according to the results of the effectiveness evaluation. At the same time, patients were stratified according to type of tumor and grade of thrombocytopenia for subgroup analysis.

**Results:** A total of 262 patients were collected and 174 patients were enrolled after PSM, 87 in the rhTPO group and 87 in the rhIL-11 group. In all patients, there were no significant differences in the platelet compliance rate, mean days of medication, median days of compliance, median highest platelet count after medication, and the median platelet count elevation before and after medication between the two groups (*p* > 0.05), but the median lowest platelet count after next-cycle cancer therapy in the rhTPO group was lower than that in the rhIL-11 group (*p* = 0.014). The subgroup analysis showed that the rhTPO group had longer mean days of medication than the rhIL-11 group in patients with hematological malignancies (*p* = 0.042), and a lower median lowest platelet count after next-cycle cancer therapy in patients with grade I/II thrombocytopenia than rhIL-11 group (*p* = 0.022), with no significant difference in other outcome indicators (*p* > 0.05). As there was no statistically significant difference in platelet compliance rate between the two groups, the cost-minimization analysis showed that the rhIL-11 group had lower treatment costs than the rhTPO group.

**Conclusion:** RhTPO and rhIL-11 showed similar effectiveness in the treatment of CTIT, but rhIL-11 was more advantageous in economic cost.

## 1 Introduction

Thrombocytopenia is a common hematologic toxicity induced by cancer therapy, causing therapy delay, dose reductions, and treatment discontinuation, negatively impacting treatment outcomes. The decrease of the platelet count put the patients at risk for bleeding, resulting in increased mortality (The Society of Chemotherapy and Committee of Neoplastic Supportive-Care (CONS) 2019; Weycker et al., 2019). Recent studies have reported that in addition to the usual radiotherapy and chemotherapy, new treatment modalities such as targeted therapy and immunotherapy can also lead to different degrees of thrombocytopenia (Gunderson et al., 2018; Liu et al., 2020). When these different treatments were combined, the incidence of thrombocytopenia was significantly higher than that of single therapy (Slamon et al., 2011; Penniment et al., 2018). The Chinese Society of Clinical Oncology defined these thrombocytopenia due to anti-tumor therapy as cancer therapy induced thrombocytopenia (CTIT), characterized by a platelet count of less than 100 × 10^9^/L in peripheral blood (Guideline Working Committee of Chinese Society of Clinical Oncology, 2022).

The treatments for CTIT mainly include platelet transfusion and the use of platelet growth factors (Kuter, 2015; Gunderson et al., 2018). Platelet infusion can rapidly and effectively improve the platelet count, but it is only used in patients with a bleeding tendency or severe thrombocytopenia due to the risk of infection and even other complications, such as platelet antibodies production or post-infusion immune response (Katus et al., 2014; The Society of Chemotherapy and Committee of Neoplastic Supportive-Care (CONS) 2019). In contrast, platelet growth factors have more advantages. Through different mechanisms of action, they can rapidly increase platelet counts, shorten the duration of thrombocytopenia, reduce platelet transfusion, and enable the intensity of anti-tumor therapy to be maintained (Tepler et al., 1996; Bai et al., 2004; Kuter, 2022). These drugs include recombinant human thrombopoietin (rhTPO), recombinant human interleukin-11 (rhIL-11), and thrombopoietin receptor agonists (TPO-RAs) such as romiplostim, eltrombopag and avatrombopag. However, TPO-RAs were initially approved for the treatment of idiopathic thrombocytopenic purpura and there is insufficient evidence for its use in CTIT, which is generally only recommended as grade II. The rhTPO and rhIL-11 approved by the China National Medical Products Administration (NMPA) for chemotherapy-induced thrombocytopenia are widely used in China as grade I options for CTIT (Guideline Working Committee of Chinese Society of Clinical Oncology, 2022).

Both rhTPO and rhIL-11 can raise the platelet count, but their effectiveness may vary and the treatment costs of the two drugs vary several times. Therefore, it is of great significance for the clinical optimal regimen therapy to evaluate their effectiveness and economics. However, existing studies of the two drugs have only been in patients receiving radiotherapy and/or chemotherapy, little information is available about their use in CTIT, and some real-world studies do not deal with confounding factors (Chen et al., 2015; Chen et al., 2019; Zhang et al., 2022). In fact, real-world data are more reflective of actual clinical use than clinical trials with strict inclusion and exclusion criteria, but there are also various confounding factors that can result in bias (Howards, 2018; Zhang et al., 2019). The propensity score matching (PSM) can be used to eliminate the influence of these confounding factors, so as to make a more reasonable comparison between the treatment group and the control group (Haukoos and Lewis, 2015). This study used PSM to eliminate confounding factors and retrospectively analyzed the effectiveness and economics of rhTPO and rhIL-11 in CTIT based on real-world data, aiming to provide references for rational drug selection in clinics.

## 2 Materials and methods

### 2.1 Data sources

We retrospectively collected the data of cancer patients treated with rhTPO or rhIL-11 for CTIT in a single cancer hospital from January 2020 to December 2021, including patients’ baseline characteristics, disease diagnosis, medications, blood test results, treatment costs, etc.

As a retrospective study, the patient’s informed consent has been exempted. This study protocol was approved by the Medical Ethics Committee of Sichuan Cancer Hospital (Ethical Approval NO. SCCHEC-02-2022-141).

### 2.2 Inclusion and exclusion criteria

Inclusion criteria included: 1) aged ≥18 years, 2) a pathological diagnosis of malignant tumor, 3) platelet count drops below 100 × 10^9^/L after cancer therapy, 4) The current cancer regimen should be continued for ≥1 cycle, 5) Eastern Cooperative Oncology Group (ECOG) performance status score ≤2.

Exclusion criteria included: 1) pregnancy or lactation, 2) missing primary clinical data, 3) use other platelet-elevating drugs or platelet infusion at the same time, 4) severe heart, lung, liver, kidney or other major organ dysfunction, 5) a history of thrombosis, sepsis or disseminated intravascular coagulation, 6) acute infection or active bleeding.

### 2.3 Treatment regimens

The rhTPO group received a daily subcutaneous injection of 300 U/kg, and the rhIL-11 group received a daily subcutaneous injection of 25–50 μg/kg.

Thrombocytopenia is classified as grade I-IV according to the grading system in the Common Terminology Criteria for Adverse Events (CTCAE) version 5.0 (November 2017), with grade I thrombocytopenia corresponding to 75 × 10^9^/L ≤ platelet count <100 × 10^9^/L, grade II corresponding to 50 × 10^9^/L ≤ platelet count <75 × 10^9^/L, grade III corresponding to 25 × 10^9^/L ≤ platelet count <50 × 10^9^/L, and platelet count <25 × 10^9^/L for grade IV. The groups were further stratified according to the type of tumor and the degree of thrombocytopenia. On the one hand, the patients were divided into solid tumor group and hematological malignancy group according to the tumor type. On the other hand, patients were divided into grade I-II CTIT group and grade III-IV CTIT group according to the grade of thrombocytopenia.

### 2.4 Evaluation indicator

Platelet compliance was defined as platelet count ≥100 × 10^9^/L or an absolute increase to ≥50 × 10^9^/L after CTIT and before the next cycle of cancer therapy (Li et al., 2018). The platelet compliance rate was the proportion of patients with platelet compliance among all patients, which was the main evaluation indicator of this study. The days of compliance were the time needed to achieve platelet compliance, and other indicators included the days of medication, the highest platelet count after medication, the platelet count elevation before and after medication, and the lowest platelet count after the next cycle of cancer therapy.

### 2.5 Cost calculation

The cost of pharmacoeconomics include direct costs, indirect costs, and hidden costs (Waugh and Mistry, 2019). The pharmacoeconomic evaluation of this study only calculated the direct costs related to patients’ treatment of diseases from the perspective of healthcare system, indirect costs, and hidden costs were not incorporated into the calculations due to measurement difficulties. Direct costs, including drug costs, routine blood test costs, and bed costs, were calculated for the total costs of treatment in this study and were uniformly expressed in monetary units (yuan), based on real-time prices in the Hospital Information System (HIS) from January 2020 to December 2021.

### 2.6 Statistical analysis

SPSS statistical software (version 25.0) was used for data analysis. Covariables such as baseline information of patients, tumor type, and platelet count before medication that may affect drug effectiveness were included, and the caliper value was set as 0.2 to achieve PSM with1:1. The measurement data were described by mean ± standard deviation (SD) or median + interquartile range (IQR), and *t*-test or Wilcoxon rank sum test was used for comparison between groups. Count data were described as absolute numbers or percentages, and comparison between groups was performed by *χ* ^*2*^ test or Fisher’s exact probability method. All statistical tests were performed using a two-sided test with a significance level of 0.05, and *p* < 0.05 was considered a statistically significant difference.

The economic evaluation was performed according to the results of the effectiveness evaluation. If there was no statistical difference in effectiveness between the two groups, the cost-minimization analysis method was used to directly compare the average cost of the two groups to determine the economy of the treatment regimen. If there was a significant difference in effectiveness between the two groups, the cost-effectiveness analysis was used and the incremental cost-effectiveness ratio (ICER) was used as an evaluation indicator. Deterministic one-way sensitivity analysis and probabilistic sensitivity analysis were used to evaluate the robustness of economic results. One-way sensitivity analysis was performed by assuming ±20% of drug costs, routine blood test costs, and bed costs. Probabilistic sensitivity analysis was performed by Bootstrap method with 1,000 repeated samplings.

## 3 Results

### 3.1 Baseline information

A total of 262 patients were collected, including 90 patients in the rhTPO group and 172 patients in the rhIL-11 group. Before PSM, there was no significant difference in sex, age, height, weight, tumor stage, cancer therapy protocol, and platelet count before cancer therapy between the two groups (*p* > 0.05). However, a statistically significant difference in tumor type and lowest platelet count after cancer therapy was identified between the two groups (*p* < 0.001). After PSM, 174 patients were included, 87 in each group. No significant difference in any of the baseline information was noted between the two groups (*p* > 0.05) (Table 1).

**TABLE 1:** Baseline information of patients before and after propensity score matching (PSM).

Variables	Before PSM		*p*	After PSM		*p*
	rhTPO (n = 90)	rhIL-11 (n = 172)		rhTPO (n = 87)	rhIL-11 (n = 87)	
Sex, n (%)			0.284			0.644
Male	38 (42.2%)	61 (35.5%)		37 (42.5%)	34 (39.1%)	
Female	52 (57.8%)	111 (64.5%)		50 (57.5%)	53 (60.9%)	
Age, Mean (±SD), years	56.31 ± 10.55	55.28 ± 11.33	0.474	56.31 ± 10.51	54.84 ± 12.09	0.393
High, Mean (±SD), cm	160.67 ± 8.53	159.28 ± 7.25	0.190	160.74 ± 8.53	160.23 ± 7.23	0.674
Weight, Mean (±SD), kg	58.98 ± 9.60	57.89 ± 8.63	0.350	58.57 ± 9.33	58.47 ± 8.81	0.940
Tumor type, n (%)			<0.001			0.527
Lymphoma	22 (24.4%)	11 (6.4%)		19 (21.8%)	10 (11.5%)	
Colorectal	12 (13.3%)	7 (4.1%)		12 (13.8%)	7 (8.0%)	
Gastric	5 (5.6%)	21 (12.2%)		5 (5.7%)	8 (9.2%)	
Esophagus	6 (6.7%)	13 (7.6%)		6 (6.9%)	6 (6.9%)	
Cervical	12 (13.3%)	37 (21.5%)		12 (13.8%)	14 (16.1%)	
Breast	5 (5.6%)	24 (14.0%)		5 (5.7%)	11 (12.6%)	
Ovarian	4 (4.4%)	13 (7.6%)		4 (4.6%)	6 (6.9%)	
Nasopharyngeal	4 (4.4%)	18 (10.5%)		4 (4.6%)	6 (6.9%)	
Lung	8 (8.9%)	9 (5.2%)		8 (9.2%)	7 (8.0%)	
Other	12 (13.3%)	19 (11.0%)		12 (13.8%)	12 (13.8%)	
Tumor stage, n (%)			0.773			0.966
I	5 (5.6%)	8 (4.7%)		5 (5.7%)	4 (4.6%)	
II	12 (13.3%)	28 (16.3%)		12 (13.8%)	13 (14.9%)	
III	37 (41.1%)	61 (35.5%)		36 (41.4%)	34 (39.1%)	
IV	36 (40.0%)	75 (43.6%)		34 (39.1%)	36 (41.4%)	
Cancer therapy protocol, n (%)			0.713			1.000
Drug therapy^a^	56 (62.2%)	103 (59.9%)		53 (60.9%)	53 (60.9%)	
Drug therapy plus radiotherapy	34 (37.8%)	69 (40.1%)		34 (39.1%)	34 (39.1%)	
Platelet count before cancer therapy, Median [IQR], ×10^9^/L	130.0 [98.0, 187.3]	124.0 [106.0, 156.5]	0.625	131.0 [99.0, 187.0]	126.0 [106.0, 167.0]	0.798
Lowest platelet count after cancer therapy, Median [IQR], ×10^9^/L	64.0 [49.0, 73.3]	71.5 [61.0, 78.0]	<0.001	64.0 [50.0, 74.0]	70.0 [56.0, 77.0]	0.085

^a^ Drug therapy indicates chemotherapy ± (targeted therapy and/or immunotherapy).

SD, standard deviation; IQR, interquartile range; rhTPO, recombinant human thrombopoietin; rhIL-11, recombinant human interleukin 11.

### 3.2 Clinical effectiveness

#### 3.2.1 Evaluation of overall effectiveness

In all patients, the compliance rate of the rhTPO group was 87.4%, higher than 81.6% in the rhIL-11 group, but the difference was not statistically significant (*p* = 0.295). There were also no significant differences between the two groups in the mean days of medication, median days of compliance, median highest platelet count after medication, and median platelet count elevation before and after medication. However, the median lowest platelet count after next cycle of cancer therapy in the rhTPO group was significantly lower than that in the rhIL-11 group [87.0(IQR, 62.0–117.5) × 10^9^/L vs. 111.0(IQR, 84.0–146.0) × 10^9^/L, *p* = 0.014] (Table 2).

**TABLE 2:** Effectiveness evaluation of two drugs.

Patient types	Drug	n	Compliance rate, n (%)	Days of medication, mean (±SD), d	Days of compliance, median [IQR], d	Highest platelet count after medication, median [IQR], ×10^9^/L	Platelet count elevation before and after medication, median [IQR], ×10^9^/L	Lowest platelet count after next cycle of cancer therapy, median [IQR], ×10^9^/L
All patients	rhTPO	87	76 (87.4%)	5.56 ± 2.52	7.0 [5.5, 12.0]	176.0 [120.5, 240.5]	105.0 [56.5, 170.0]	87.0 [62.0, 117.5]
	rhIL-11	87	71 (81.6%)	4.77 ± 2.85	7.0 [4.0, 15.0]	164.0 [135.0, 249.0]	101.0 [71.0, 177.0]	111.0 [84.0, 146.0]
*p*			0.295	0.053	0.505	0.708	0.622	0.014
Solid tumor group	rhTPO	67	58 (86.6%)	5.58 ± 2.55	7.0 [5.0, 10.0]	174.0 [123.0, 225.0]	105.0 [58.0, 162.0]	87.0 [68.0, 118.0]
	rhIL-11	76	61 (80.3%)	4.92 ± 2.97	7.0 [4.0, 15.0]	164.0 [135.0, 237.5]	101.0 [72.0, 167.5]	114.0 [85.5, 149.0]
*p*			0.314	0.158	0.910	0.771	0.658	0.055
Hematological malignancy group	rhTPO	20	18 (90.0%)	5.50 ± 2.48	11.0 [6.0, 21.5]	191.0 [118.8, 334.8]	114.5 [48.3, 266.5]	68.5 [39.5, 121.3]
	rhIL-11	11	10 (90.9%)	3.73 ± 1.62	6.0 [4.8, 20.5]	174.0 [132.5, 335.5]	103.0 [58.5, 267.3]	98.5 [71.5, 133.5]
*p*			1.000	0.042	0.456	0.934	0.918	0.121
I/II thrombocytopenia group	rhTPO	68	61 (89.7%)	5.29 ± 2.52	7.0 [5.0, 12.3]	172.5 [119.8, 229.8]	96.5 [52.5, 151.8]	87.0 [62.8, 113.5]
	rhIL-11	74	59 (79.7%)	4.62 ± 2.88	7.0 [4.0, 15.0]	165.0 [141.0, 226.0]	102.0 [68.0, 158.0]	112.0 [89.0, 138.0]
*p*			0.101	0.142	0.546	0.604	0.590	0.022
III/IV thrombocytopenia group	rhTPO	19	15 (78.9%)	6.53 ± 2.34	7.0 [6.0, 10.0]	194.0 [153.0, 242.0]	147.0 [105.0, 195.0]	82.0 [45.0, 138.0]
	rhIL-11	13	12 (92.3%)	5.62 ± 2.66	6.5 [5.3, 12.3]	138.0 [120.8, 294.5]	91.5 [86.0, 246.0]	83.5 [65.8, 161.8]
*p*			0.598	0.315	0.692	0.744	0.788	0.379

SD, standard deviation; IQR, interquartile range; rhTPO, recombinant human thrombopoietin; rhIL-11, recombinant human interleukin 11.

#### 3.2.2 Effectiveness evaluation of patients with different tumor types

In a total of 143 patients with solid tumors, no statistical difference was found in the compliance rate between the two groups (86.6% vs. 80.3%, *p* = 0.314). The same results were shown in the mean days of medication, median days of compliance, median highest platelet count after medication, median platelet count elevation before and after medication, and median lowest platelet count after next cycle of cancer therapy between the two groups.

Data from 31 patients with hematological malignancies were analyzed and the compliance rates of the two groups were 90.0% and 90.9%, respectively, with no statistical difference (*p* = 1.000). In addition, no statistical differences were found for other evaluation indicators, except for the rhTPO group, which had significantly longer days of medication than the rhIL-11 group [(5.50 ± 2.48) d vs. (3.73 ± 1.62) d, *p* = 0.042] (Table 2).

#### 3.2.3 Effectiveness evaluation of patients with different grades of thrombocytopenia

A total of 142 patients had grade I-II CTIT. No significant difference in the compliance rate was noted between the two groups (89.7% vs. 79.7%, *p* = 0.101). There were also no significant differences in the mean days of medication, median days of compliance, median highest platelet count after medication, and median platelet count elevation before and after medication between the two groups. However, the median lowest platelet count after next cycle of cancer therapy in the rhTPO group was significantly lower than that in the rhIL-11 group [87.0(IQR, 62.8–113.5) ×10^9^/L vs. 112.0(IQR, 89.0–138.0) ×10^9^/L, *p* = 0.022].

There were 32 patients with grade III-IV CTIT. The compliance rate was 78.9% in the rhTPO group and 92.3% in the rhIL-11 group, and the difference was not statistically significant (*p* = 0.598). There were no significant differences in other outcome indicators between the two groups (Table 2).

### 3.3 Economic evaluation

#### 3.3.1 Cost-minimization analysis

In this study, there was no statistical difference in the compliance rate between the two groups (*p* > 0.05), so the cost-minimization analysis was conducted, and the regimen with a lower average cost was the preferred regimen.

The results showed that for all patients, the total treatment costs of the rhIL-11 group was 1294.33 yuan, and that of the rhTPO group was 5518.27 yuan. The rhTPO group was 4223.94 yuan higher than the rhIL-11 group, indicating the rhIL-11 group was more economical. The cost-minimization analysis of different types of patients showed that the total costs of treatment in the rhIL-11 group were lower than that in the rhTPO group in patients with solid tumors, hematological malignancies, I/II CTIT, and III/IV CTIT. It can be seen that the rhIL-11 group was more cost-advantageous (Table 3).

**TABLE 3:** Cost-minimization analysis.

Patient types	Drug	n	Drug costs per patient (yuan)	Routine blood test costs per patient (yuan)	Bed costs per patient (yuan)	Total costs per patient (yuan)	Cost difference per patient (rhTPO minus rhIL-11) (yuan)
All patients	rhTPO	87	5157.10	73.45	287.72	5518.27	+4223.94
	rhIL-11	87	991.78	65.52	237.03	1294.33	
Solid tumor group	rhTPO	67	5112.81	70.52	293.37	5476.70	+4147.95
	rhIL-11	76	1018.37	66.12	244.26	1328.75	
Hematological malignancy group	rhTPO	20	5305.50	83.25	268.80	5657.55	+4600.99
	rhIL-11	11	808.11	61.36	187.09	1056.56	
I/II thrombocytopenia group	rhTPO	68	5009.12	71.47	278.56	5359.15	+4114.65
	rhIL-11	74	949.52	65.68	229.30	1244.50	
III/IV thrombocytopenia group	rhTPO	19	5686.74	80.53	320.53	6087.80	+4509.76
	rhIL-11	13	1232.34	64.62	281.08	1578.04	

rhTPO, recombinant human thrombopoietin; rhIL-11, recombinant human interleukin 11.

#### 3.3.2 One-way sensitivity analysis

**FIGURE 1:**
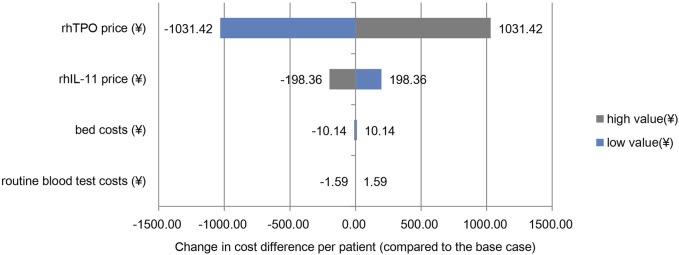
One-way sensitivity analysis. Note: the base case value is the cost difference per patient between the two groups of ¥4,223.94.

The analysis results at ± 20% of the assumed variable price were consistent with the economic analysis results, and the rhIL-11 group was more economical. As shown in the Tornado diagram (Figure 1), the price changes of rhTPO and rhIL-11 had the greatest impact on the cost difference between the two groups, and the changes in routine blood test costs and bed costs had little effect on the results. When the price of rhTPO increased or decreased by 20%, the cost difference between the two groups ranged from 3192.52 yuan to 5255.36 yuan. When the price of rhIL-11 increased or decreased by 20%, the cost difference between the two groups ranged from 4025.58 yuan to 4422.30 yuan.

#### 3.3.3 Probabilistic sensitivity analysis

The bootstrap method was used to perform 1000 repeated samples with return in the rhTPO group and rhIL-11 group, and the confidence intervals were calculated for the total costs of the two groups (Table 4). The results showed that the confidence intervals of the total costs of the two groups did not overlap, indicating that the difference between the two groups was significant, suggesting that the rhIL-11 group had lower total costs and was more economical than the rhTPO group. The results of the bootstrap analysis were consistent with the results of the economic analysis, indicating that the economic evaluation results were more robust.

**TABLE 4:** Bootstrap analysis.

Group	Total costs per patient (yuan)	95%CI
rhTPO	5518.27	(5016.28, 6168.88)
rhIL-11	1294.33	(1153.04, 1453.52)

CI, confidence interval; rhTPO, recombinant human thrombopoietin; rhIL-11, recombinant human interleukin 11.

## 4 Discussion

CTIT is a relatively intractable hematologic toxicity, its incidence is related to tumor type, therapy modality, and whether or not combination therapy is used (Liebman, 2014; Oncology Support Rehabilitation Therapy Group of Oncology Branch of Chinese Medical Association, 2021). RhTPO and rhIL-11, as recommended grade I drugs for the treatment of CTIT, are widely used in clinical practice in China. rhTPO is an endogenous cytokine that stimulates the growth and differentiation of megakaryocytes. It stimulates all stages of megakaryocyte production, including the proliferation of precursor cells and the development and maturation of megakaryocytes (Begley and Basser, 2000; Hitchcock and Kaushansky, 2014). rhIL-11 induces the maturation and differentiation of megakaryocytes by stimulating the proliferation of bone marrow hematopoietic stem cells and megakaryocyte progenitors, which then increases platelet production *in vivo* (Bhatia et al., 2007; Vadhan-Raj, 2009). Both rhTPO and rhIL-11 can elevate platelet count to normal levels, but their effectiveness may vary and their prices vary widely, so it is necessary to evaluate their effectiveness and economy.

In this study, cases with balanced differences between groups were enrolled using PSM and then analyzed. The effectiveness evaluation results showed that the compliance rate in the rhTPO group was higher than that in the rhIL-11 group, but the difference was not statistically significant (87.4% vs. 81.6%, *p* = 0.295), nor was the compliance rate in the subgroup analysis, which was similar to the results of some studies (Chen and Yang, 2016; Li et al., 2018; Chen et al., 2019). However, these results were inconsistent with the results of some other studies (Chen et al., 2019; Zhang et al., 2022), which may be due to the different times of evaluating the outcome indicators. In addition, there were no significant differences in the median days of compliance, median highest platelet count after medication, and the median platelet count elevation before and after medication between the two groups in the overall patients and subgroup analyses, but the mean days of medication of rhTPO group were significantly longer than that of rhIL-11 group in patients with hematological malignancies [(5.50 ± 2.48) d vs. (3.73 ± 1.62) d, *p* = 0.042]. In fact, in both the overall and subgroup analyses of this study, the days of medication in the rhTPO group were longer than that in the rhIL-11 group, but the difference was only statistically significant in patients with hematologic malignancies. This may be due to the fact that the patients in the rhTPO group had lower baseline platelet values before platelet-raising therapy, and it may also be related to the characteristics of the hematological diseases, resulting in longer medication days (Shahrabi et al., 2018).

This study also compared the two drugs in terms of their effect on platelets after the next cycle of cancer therapy. The result showed that in all patients and patients with grade I/II thrombocytopenia, the median lowest platelet count after the next cycle of cancer therapy in the rhTPO group was significantly lower than that in the rhIL-11 group (all *p* < 0.05). This result was similar to the previous study, which also found a greater effect of platelets after next-cycle cancer therapy with the rhTPO (Li et al., 2018). We speculated this result may be related to the mechanism of action and half-life of the two drugs. RhTPO acts on the whole process from hematopoietic stem cells to platelet generation, preceding the action stage of rhIL-11 (Taguchi et al., 2001). Additionally, the half-life of rhTPO is approximately 40 h, longer than that of rhIL-11 at 6.9 h. These reasons may lead to bone marrow remaining activated until the next cycle of cancer therapy, resulting in newborn megakaryocytes being killed by a new cycle of cancer treatment, and failing to differentiate into mature platelets. Furthermore, in terms of economic evaluation, as there was no statistical difference in the compliance rate between the two groups, the cost-minimization analysis showed that the total costs of the rhIL-11 group were lower than that of the rhTPO group, and rhIL-11 had more economically advantageous.

This study was the first to evaluate the effectiveness and economy of rhIL-11 and rhTPO in patients with CTIT, which is different from previous studies on patients treated with radiotherapy and/or chemotherapy alone and can provide a reference for drug selection of thrombocytopenia caused by diversified cancer treatment modalities. However, this study was a retrospective analysis, although PSM was used to eliminate confounding factors between the two groups, it still cannot be equated with randomized controlled trials and there may be other influencing factors such as diet and combined medication that may affect the results. In addition, in terms of adverse reactions, the nature of retrospective studies made it difficult to distinguish whether the adverse reactions of patients were caused by cancer treatment or platelet-raising drugs. According to previous clinical studies, the common adverse reactions of rhIL-11 include fatigue, fever, edema, tachycardia, allergic reaction, transient anemia, conjunctival congestion, etc. The common adverse reactions of rhTPO include fever, chills, general discomfort, fatigue, knee pain, headache, dizziness, elevated blood pressure, etc. Most of these adverse reactions can be recovered without any measures (Hou et al., 2015; Liu et al., 2018). Therefore, the total costs of this study did not include the treatment costs of adverse reactions, which may affect the results of the pharmacoeconomic evaluation. Furthermore, this study was a single-center study and the sample size was small, which had limitations and may lead to deviations in the findings from the actual situation.

## 5 Conclusion

In summary, both rhTPO and rhIL-11 could promote the recovery of platelet count in CTIT patients with similar effectiveness, but rhIL-11 had more advantages in economic cost. However, this study had some limitations that might affect the results of the study. Therefore, the results need to be further demonstrated in more well-designed randomized controlled trials or real-world research with larger sample sizes from multiple centers.

## Data Availability

The original contributions presented in the study are included in the article/supplementary material, further inquiries can be directed to the corresponding author.
